# Population-level toggling of T cell immune escape at human leukocyte antigen anchor residues in SARS-CoV-2 Spike proteins, in an ethnically diverse population region

**DOI:** 10.1371/journal.pcbi.1013261

**Published:** 2025-07-21

**Authors:** Nobubelo K. Ngandu, Burtram C. Fielding, Peter van Heusden, Kuhle Mcinga, Kriheska Francis, Gordon Harkins

**Affiliations:** 1 HIV and other Infectious Diseases Research Unit, South African Medical Research Council, Cape Town, South Africa; 2 South African National Bioinformatics Institute (SANBI), University of the Western Cape, Cape Town, South Africa; 3 Molecular Biology and Virology Research Laboratory, Department of Medical Biosciences, Faculty of Natural Sciences, University of the Western Cape, Cape Town, South Africa; 4 Department of Microbiology, Faculty of Science, Stellenbosch University, Stellenbosch, South Africa; University of Auckland, NEW ZEALAND

## Abstract

There is currently limited understanding of the severe acute respiratory syndrome coronavirus-2 (SARS-CoV-2) adaptation to the human leukocyte antigen (HLA) proteins which mediate CD8 (HLA-I) and CD4 (HLA-II) T cell immune responses. We investigated population-level T cell immune escape in SARS-CoV-2 Spike protein at amino acid binding positions (the anchor motifs) preferred by the highly restrictive peptide binding grooves of the HLA. SARS-CoV-2 Spike protein sequences isolated in South Africa from January 2020 until June 2022, were used. All possible 9-mer and 15-mer peptides in the sequence alignment were scanned for matches to HLA-I and HLA-II anchor motifs, respectively. Peptide positions with matched anchor motifs and ≥1% mismatched sequences were investigated for immune escape using immunoinformatic prediction methods and directional evolution along the phylogenetic tree. Toggling of short-lived immune escape mutations at HLA-I anchor motifs was observed in 17 peptides across Spike. Eight of these overlapped with HLA-II escape mutations. Six mutations were related to zoonotic adaptation. All 17 sites were under significant directional evolution along the phylogenetic tree, and 16/17 are within published confirmed or inferred T cell epitopes. Immune escape predictions for HLA- A*66:01/A*68:01 were common (n = 7/17). HLA- A*02:05, A*03:01, B*07:02, B*08:01, B*58:01, DRB1*04:01 and DQA1*01:02-DQB1*06:02 were each associated with at least two escape mutations. This immunoinformatic prediction of T cell immune escape at HLA anchor motifs: (i)shortlisted potentially understudied population-specific HLA and immune escape (ii)revealed a footprint of underlying toggling of short-lived immune escape mutations, and (iii)has potential to cost-effectively guide pre-clinical research questions on the inclusion of partially conserved but dominant epitopes in vaccine immunogens.

## Author summary

During a T cell immune response, protein molecules known as Human leukocyte antigens (HLA) bind to foreign pathogen peptides and present these to T cells. The genetic diversity of HLA within and between populations enables immune responses against a wide range of infecting pathogens. Each HLA has pockets where binding affinity to the pathogen peptide is strongest and only certain unique amino acids (known as an ‘anchor motifs’) are required. Viruses can mutate amino acids at the anchor motifs as a mechanism to prevent HLA binding and hence escape attack by the T cell immune response (i.e., immune escape). Here we investigated immune escape at anchor motifs in SARS-CoV-2 Spike peptides of viruses circulating in South Africa during 2020–2022. We found seventeen Spike peptides with immune escape at anchor motifs for HLA which are present in high frequency in at least one major ethnic group in South Africa. Short-lived but repeated episodes (i.e., ‘toggling’) of immune escape were observed. All mutations are known variants of concern, and 16/17 peptides are previously confirmed T cell targets, affirming the importance of this computational immunology analysis in revealing and short-listing important immune responses in a defined population, for further investigation in pre-clinical research.

## Introduction

Data on the role of Human leukocyte antigen (HLA) alleles in shaping mutations of the severe acute respiratory syndrome coronavirus-2 (SARS-CoV-2) viruses that enable evasion of HLA-epitope binding and hence escape from HLA-mediated T cell immune responses are currently limited. The human genes encoding HLA alleles are highly polymorphic [1–3]. The binding groove of each allelic serotype preferably binds a unique set of amino acid combinations (the anchor motifs) within epitopes at high affinity to successfully prime the T cell immune response pathway [4]. Therefore, the selective characteristic of the HLA groove is a major determinant of which epitopes are successfully presented to CD8 T cells (HLA class I, HLA-I) and to CD4 T cells (HLA class II, HLA-II).

HLA-I alleles commonly bind 9-mer peptides with some allowing a range of 8–12 mers [5–7]. The amino-acid positions forming the anchor motifs are typically in the second and C-terminal position of a peptide [4,5,8]. HLA-II binding peptides are mostly 15-mers long but can be longer. The HLA-II core anchor motif pattern is complex and exhibits several anchors spanning a 9-mer starting between positions 3–5 of the N-terminal flanking loop [4].

Point mutations within anchor motifs is a common mechanism used by viruses to evade HLA binding and escape the host T cell immune response [9,10]. Thus, anchor motifs have been used widely to understand how viruses, such as in Human Immunodeficiency virus (HIV) and human papilloma virus (HPV), adapt to dominant and immunogenic T cell immune responses and how differences in HLA allele frequencies between ethnic populations introduce different epitope mutations in locally circulating viruses [11–14]. Given the observations that SARS-CoV-2 variants emerge and persist variably by geographic region, the dominant genetic characteristics of the host populations become eminent as contributing factors to virus evasion of dominant immune responses. The human host HLA compliment can also provide clues to adaptive mutations during zoonotic jumps [15].

Given the high cost of laboratory assays, Bioinformatics methods are invaluable in analyzing the accumulated SARS-CoV-2 large sequence data and produce a streamlined list of the most probable dominant immune response targets and escape patterns for confirmation in laboratory pre-clinical studies. Other bioinformatics prediction studies have reported lists of predicted and confirmed SARS-CoV-2 T cell epitopes [16–18] and this study aims to address important gaps which have received limited attention. These include limited attention to differences in HLA allele genotype frequencies between population-level ethnicity, which in many cases can be defined by geographic boundaries; strong bias towards globally dominant and/or well-studied HLA alleles; little or nuanced attention to the heterogeneity of HLA-binding requirements and hence binding affinity between amino acid positions within an epitope; and limited population-level investigations of mutations which specifically occur to escape HLA binding.

In this study we hypothesized that surveillance of amino acid anchor motifs preferred by the highly restrictive peptide binding grooves of HLA molecules can highlight population level footprints of the most important SARS-CoV-2 T cell immune escape variants associated with perpetuating infection spread. Therefore, we aimed to develop an Immunoinformatic (i.e., investigation of immunology mechanisms using computational methods) analysis pipeline which prioritizes HLA restricted preferences at anchor motifs of peptides to predict the most important T cell immune escape mutations associated with dominating HLA allele activity within a defined geographic population. This study will contribute to the understanding of how HLA genotypes influence SARS-CoV-2 genetic evolution patterns and specifically highlight important HLA and SARS-CoV-2 T cell epitopes for consideration in pre-clinical vaccine studies targeted at inducing T cell immune responses.

## Methods

### Ethics statement

Secondary anonymized open-source data were used. The study was approved by the South African Medical Research Council Human Ethics Committee in March 2022 (protocol number: EC006-2/2022).

### SARS-CoV-2 protein sequence data

The SARS-CoV-2 protein sequence alignment (‘allprot1020.tar.xz’) and corresponding metadata, were downloaded from GISAID (https://gisaid.org/) in October 2022, S1 Table [19–21]. Data isolated from South Africa were extracted and low quality sequences excluded using the Nextclade quality control tool [22]. The Spike protein region was extracted and realigned against the Wuhan-Hu-1 (Wuhan-1 or WIV04) SARS-CoV-2 reference sequence(GenBank accession number NC_045512), using MEGA (Molecular Evolutionary Genetics Analysis v11.0.14) [23]. Any sequences with ambiguity in at least 10% of amino acid positions were further excluded and the remaining alignment split into Coronavirus Disease 2019 (COVID-19) infection waves (weeks of peak infection spread) and inter-wave periods as follows: pre-wave1 (all until 30-May-2020), wave-1 (31-May-2020 to 29-August-2020), pre-wave2 (30-August-2020–28-November-2020), wave-2 (29-November-2020–6-February-2021), pre-wave3 (7-February-2021 to 15-May-2021), wave-3 (16-May-2021 to 25-September-2021), pre-wave 4 (26-September-2021–27-November-2021), wave-4 (28-November-2021–22-January-2022), pre-wave 5 (23-January-2022–16-April-2022) and wave-5 (17-April-2022–18-June-2022) [24–26]. Sequences containing amino acid insertions with respect to Wuhan-1 were analyzed separately to clearly identify presence of anchor motifs with and without insertions (S1 Table).

Sarbecovirus strains isolated from bat hosts since 2008, were used to estimate the ancestral population for SARS-CoV-2. The sequence data were obtained from an online source: Visualizing selection analysis results for evolution of nCOV (Nov 2021 update)/ Sergei Pond/ Observable (observablehq.com) and previously used to estimate and understand SARS-CoV-2 origins and ancestral evolution [27]. The nucleotide sequences were aligned in-frame to Wuhan-1 using the MAFFT v7.520 software [28,29]. Sequences with poor alignment quality were excluded and the final alignment had 130 sequences (accession numbers in S2 Table). The nucleotide alignment was translated, stop codons were replaced with gaps and any codon insertions relative to Wuhan-1 were deleted.

### HLA anchor residue motifs

The HLA-I and HLA-II anchor motifs data were downloaded from the Los Alamos National Laboratory (LANL) Immunology database motif scanner (https://www.hiv.lanl.gov/content/immunology/motif_scan/motif_help.html#Motif_Scan_Help) on 25 March 2022. The anchor motifs initially collated by Marsh et al 2000, Rammensee et al 1999 and Sette et al 1999, are continuously compiled and updated from various sources publishing HLA binding to different pathogenic peptides [3,30,31]. Four-digit resolution HLA-I alleles with 9-mer anchor motifs containing at least two defined anchor positions were shortlisted (N = 137, S3 Table) and used for predicting HLA-I associated immune escape. Octamer, nonamer and decamer HLA-II anchor motifs were shortlisted (N = 45, S4 Table). The octamer, nonamer and decamer anchor motifs were extended to 15-mers to fit the minimum requirement of the HLA-II binding groove, by allowing 3-amino acid long C-terminal flanking loop and a 4, 3 or 2 amino acid long N-terminal flanking loop, respectively (http://www.syfpeithi.de/bin/MHCServer.dll/Info.htm#specific%20informatin) [8,32,33].

### Prediction of escape mutation at anchor motifs

**Fig 1:**
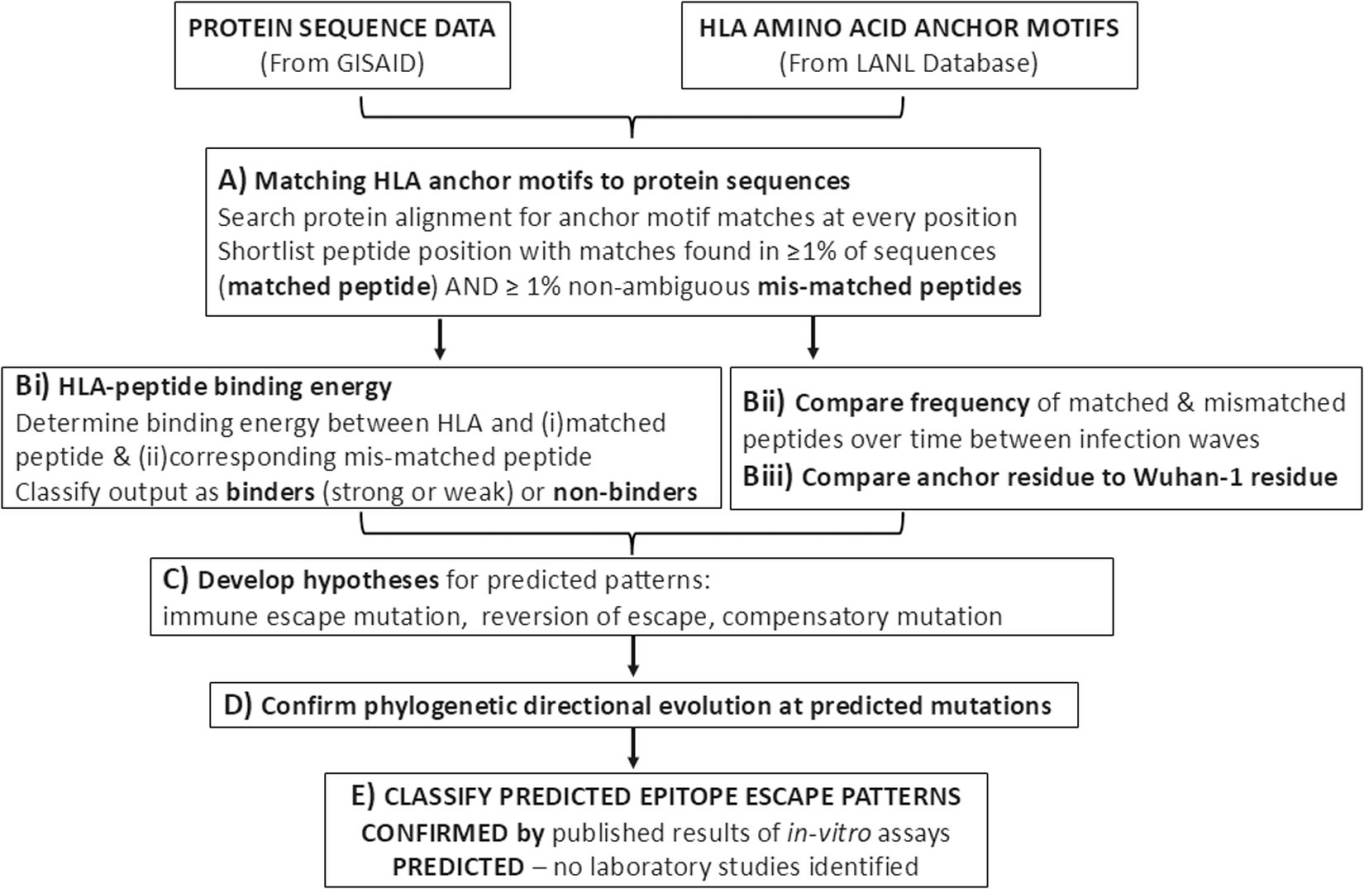
An overview of the data analysis pipeline used.

An overview of the data analysis pipeline is shown in Fig 1. The prediction of immune escape mutations at anchor motifs involved *four steps*. [1] Fig 1A: Searching for all possible (starting from every amino acid position) peptides along each sequence alignment, that matched each anchor motif, using a custom python script (python codes in S1 Code and S2 Code). Peptide positions with anchor motif matches in ≥1% of the sequences and ≥1% mismatches without ambiguous characters were shortlisted as potential HLA escaping targets; [2] *Fig 1Bi*: the binding affinity of matched-peptides and corresponding mismatched peptides to the respective HLA-I or HLA-II was determined using the NetMHCpan v4.1b and NetMHCIIpan v4.0 software, respectively [34,35]. HLA-peptide pairs with available binding affinity results (i.e., strong, weak or non-binders) were extracted; [3] *Fig 1Bii:* peptide positions predicted in at least two infection waves alignments (to estimate dominant immune responses) were analyzed further; [4] hypotheses of predicted immune escape patterns were synthesized from binding affinity, peptide frequency over time and whether the matched anchor residue was a Wuhan-1 or variant amino acid residue.

**Fig 2:**
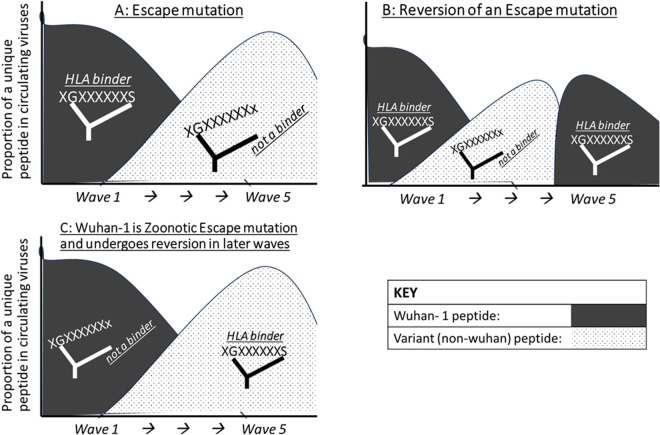
Illustrations for hypotheses of different immune escape patterns. The illustrations shown are for: an immune escape **(A)**, reversion of an immune escape **(B)**, and reversion of a zoonotic escape mutation **(C)**. Grey curve = sequences with wuhan-1 peptide and dotted curve = sequences with newly emerged peptide with variant residue at the HLA anchor position.

The classifications were: *escape mutation*- an anchor motif matched-peptide is an HLA binder with a Wuhan-1 anchor residue, while the corresponding mis-matched peptide (emerged variant at same anchor) is a non-binder to the same HLA and replaces the Wuhan-1 anchor residue in the population (Fig 2A). If the frequencies of the two residues change in the opposite direction in later infection waves, then a *reversion of the escape* is inferred (Fig 2B). *Zoonotic escape mutation*- the same pattern as escape mutation except the anchor motif matched HLA binder peptide has an emerged variant residue at the anchor while the mismatched non-binder is a Wuhan-1 residue. A *reversion of a zoonotic escape* mutation would be a decreasing frequency of the mismatched non-binder Wuhan-1 peptide being replaced by the increasing emerged HLA binder variant residue (Fig 2C); *compensatory mutation*- a newly emerged amino acid variant at a non-anchor position within or adjacent to the peptide and its frequency changes coincide with some changes in the predicted escape mutation; *toggling*- general term for several short-lived amino acid changes which could be escape mutations, reversions or compensations. Peptide binders but lacking the known HLA motif were noted as previously unpublished potential new anchor-residues. INDELS were investigated for potential immune escape or compensation mechanisms.

### Phylogenetic inference of directionally evolving sites

The direction of evolution on the phylogeny of protein sequences was determined to confirm whether the predicted immune escaping sites are under significant selective pressure and whether the predicted escape mutations are significant evolutionary targets. A Bayesian framework model FADE (FUBAR Approach to Directional Evolution), run within the HyPhy package v2.5.49 (Hypothesis testing using Phylogenies), was used to determine amino acid sites undergoing directional evolution (i.e., sites where there is strong preferential evolution towards a specific amino acid residue, and the residues from which the evolutions tends to occur) [36–38]. Directional evolution was determined within the SARS-CoV-2 phylogeny and separately for the combined SARS-CoV-2/sarbecovirus phylogeny to understand evolutionary targets during infection spread in South Africa and during zoonotic spillover, respectively. The detailed methods are in S1 Text.

### HLA allele frequency data

Information on population-level frequency of HLA alleles was mostly obtained from the Allele Frequency Net Database (AFND), last updated in 2019 (The Allele Frequency Net Database [HLA top 10 frequencies] (allelefrequencies.net) [39,40]. Literature search was conducted for HLA whose frequency in South Africa is not yet included in the AFND.

### Confirmed T cell epitopes

The IDEB database (https://www.iedb.org) and rapid literature searches were used to identify laboratory confirmed T cell epitopes [41].

## Results

### Predicted immune escape patterns at HLA anchor residue motifs

Dominant immune escape associated with HLA-I binding to anchor motifs was predicted in 17 peptides across the Spike protein. All 17 anchor positions with predicted escape were under significant directional evolution (Bayes Factor>100). Six of these (N440K, N501Y, A701V, G769V N856K and L981F) were predicted as zoonosis associated escape. Among these six sites, either the predicted zoonotic escape variant only (3/6), the emerged HLA-binding residue (i.e., reversion of the zoonotic escape) only (1/6) or both residues (2/6) were identified as zoonotic directional evolution targets (i.e., on the SARS-CoV-2/sarbecoviruses phylogeny). The direction of predicted escape mutations in 7/11 remaining peptides was consistent with directional evolution targets identified in the South Africa SARS-CoV-2 phylogeny, while in the other four sites, the evolutionary pressure was stronger away from the predicted escape variant. Compensatory mutations were predicted in 9/17 peptides, and some were directional evolution targets. A total of eight peptides were predicted to have HLA-II associated dominant escape mutations at anchor motifs and all eight overlapped with HLA-I predicted peptides. Overall, 7/17 peptides had immune escape associated with HLA A*66:01/A*68:01. Other HLA associated with at least two escape peptides were A*02:05 (3/17), B*58:01 (2/17), B*07:02 (2/17), B*08:01 (2/17 one of these being linked to A*66:01/A*68:01), and A*03:01 (3/17 all linked to A*66:01) for HLA-I and DRB1*04:01 (2/8) and DQA1*01:02-DQB1*06:02 haplotype (3/8) for HLA-II.

### HLA A*66:01/A*68:01 associated predicted immune escape patterns

**Fig 3:**
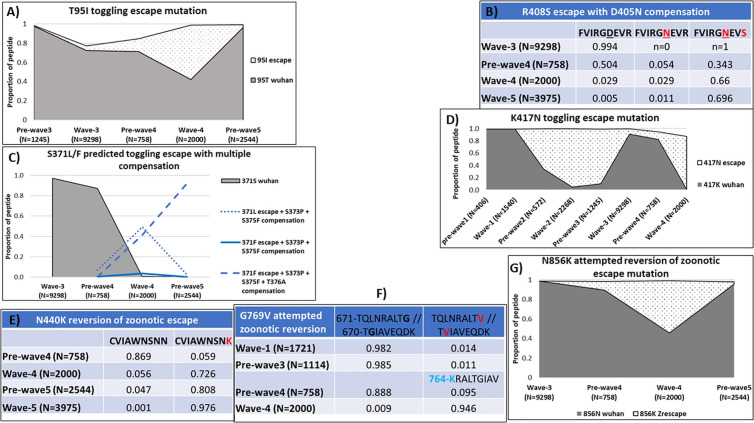
Escape patterns associated with HLA- A*66:01/A*68:01. The proportions are shown as follows in all graphs: Wuhan residue = solid grey area; predicted escape variant = dotted area; predicted compensatory mutations = different styles of line graphs; escaping anchor position = bold-face font residue in the peptide; compensatory position = underlined residue in the peptide. **A)** Toggling between predicted escape and the Wuhan residue at second anchor position of the HLA- A*68:01 binding peptide ’94-S**T**EKSNIIR-102’. The escape variant 95**I** also binds to the same HLA allele. DRB1*04:01 is associated with same T95I escape in 90-VYFAS**T**EKS-98 (15mer = 87-NDGVYFAS**T**EKSNII). **B)** HLA-A*66:01 and A*68:01 drive escape at 408, compensation 405N alone fails, but compensation with escape increase. DQA1*01:02-DQB1*06:02 binds the emerged 408S escape with 405N compensation. **C)** Proportions of predicted toggling escape at second anchor position of 370-NSASFSTFK-378 peptide binder to HLA- A*03:01 and A*66:01, with compensatory mutation sites 373 and 375. The escape mutation S371F dominates with additional compensation of T376A. **D)** Toggling between predicted escape and the Wuhan residue at C-term anchor position for the HLA- A*03:01 and A*66:01 binding peptide ’409-QIAPGQTG**K**-417’. The escape variant 417N is not a binder. **E)** N440K predicted reversion of zoonotic immune escape. The variant 440K is a binding C-term anchor motif for HLA- A*03:01/ A*66:01/ A*68:01. **F)** HLA-A*02:06/ A*66:01/ A*68:01 bind to the 769V variant. Wuhan likely zoonotic escape, with sporadic reversion attempts. DQA1*01:02-DQB1*06:02 also binds to 769V variant but DRB1*04:01 binds to Wuhan in overlapping 764-**N**RALT**G**IAV-772 (15mer = 761-TQLNRALTGIAVEQD). N-terminal N764K anchor mutation also observed and both binders to HLA-II. **G)** N856K attempted reversion of zoonotic immune escape at a cryptic anchor motif for HLA-B*08:01. The variant peptide ‘852-AQKF**K**GLTV-860’ is a strong binder but the Wuhan peptide is not a binder. The variant is also a C-term anchor for an overlapping A*66:01 binding peptide 848-DLICAQKF**K**-856.

The predicted immune escape mutations associated with HLA A*66:01/A*68:01 were T95I, S371L/F, R408S, K417N, N440K, G769V and N856K, the latter three predicted for zoonotic adaptation (i.e., escape enabling zoonosis spillover) (**Fig 3**). The T95I mutation is in the second position anchor of the HLA-A*68:01 binding peptide 94-S**T**EKSNIIR-102. Both residues are binders to the same allele. The Wuhan T95 is also a binding anchor to DRB1*04:01 in “90-VYFAS**T**EKS-98” (15-mer = 87-NDGVYFAS**T**EKSNII-101). The escape variant 95I emerged at low frequency at the end of wave-2. Toggling between the two residues followed between wave-3 and wave-5 (**Fig 3A**). Toggling was supported by directional evolution showing both residues being significant evolutionary targets. Several short-lived potential compensatory mutations emerged within the peptide during wave-3 at low frequencies (96D, 97N, 98F, 102G/S). The 97N and 98F were directional evolution targets. In the A*68:01 and A*66:01 binding peptide 400-FVIRGDEV**R**-408, a non-binder variant emerged during wave-3 at the C-terminal anchor position R408S (**Fig 3B**). This potential escape mutation increased to nearly 70% by wave-5 even though predicted to bind HLA-II DQA1*01:02-DQB1*06:02 allele. A potential compensatory mutation D405N co-emerged but remained at <5% throughout. The Wuhan residues at both sites were the directional evolution targets.

HLA A*66:01 and A*03:01 were associated with toggling escape mutation S371L/F, escape mutation K417N and N440K zoonotic reversion. The Wuhan residue and both predicted escape mutations (S371L/F) at second anchor position in 370-NSASFSTFK-378 bind to both alleles. The Wuhan residue S371 was completely replaced by equal proportions of the two variants during wave-4 coinciding with emergence of two compensatory mutations S373P and S375F (Fig 3C). The variant residue ‘L’ was short-lived while residue ‘F’ was sustained with emergence of a third compensatory mutation T376A. All emerged variants bind to several B*44 and B*15 alleles in C-terminal anchors of overlapping peptides (S5 Table). The Wuhan residues in the escape and compensatory positions were the directional evolution targets. Toggling between the predicted escape and the Wuhan residue at C-term anchor position, K417N in the predicted binding peptide’409-IAPGQTGK-417’ was observed between wave-1 through to wave-4 (Fig 3D). The escape variant 417N is not a binder to these alleles and was the directional evolution target. In a predicted reversion of a zoonotic immune escape N440K, the latter residue increased from 5% in wave-3 to >80% by wave-4 and nearly 100% by wave-5 (Fig 3E). The variant residue K is a C-terminal anchor binder for HLA Alleles A*03:01, A*66:01 and A*68:01 in the 331-CVIAWNSNK-440 peptide. The Wuhan residue N440 is not a binder and was a significant zoonotic directional evolution target.

The G769V and N856K mutations were attempted reversions of zoonotic escape. The 769V variant, a binder in overlapping C-term and second-anchor positions for HLAs A*02:06/ A*02:14 in 761-TQLNRALT**V**-769 and HLAs A*66:01/A*68:01 in 768-T**V**IAVEQDK-776, respectively, appeared at low frequencies (<2%) during wave-1 and again during pre-wave-3 (**Fig 3F**). This variant also binds to the HLA-II DQA1*01:02-DQB1*06:02 haplotype and was a zoonotic directional evolution target. The Wuhan G769 is a non-binder to these alleles and dominated the circulating strains at >98%. An overlapping DRB1*04:01 binding peptide 764-**N**RALT**G**IAV-772 (15mer = 761-TQLNRALTGIAVEQD) was observed with a N-terminal anchor mutation N764K where the variant residue was also a binder. This mutation appeared immediately after the G769V mutation was lost and could be a preferred compensation. The N856K zoonosis adaptive mutation is a binder to HLA- A*66:01 in 848-DLICAQKF**N**-856, and to B*08:01 overlapping cryptic motif ‘xx[K(R)]x[K(RH)]xxxx’ in 852-AQKF**N**GLTV-860. The Wuhan N856 residue is a non-binder to both alleles and was the zoonotic directional evolution target. The 856K variant emerged during wave-3, occupied >50% of circulating viruses by the peak of wave-4 before declining rapidly to less than 3% before the peak of wave-5 (**Fig 3G**).

### Other immune escape patterns predicted during the South Africa infection waves

**Fig 4:**
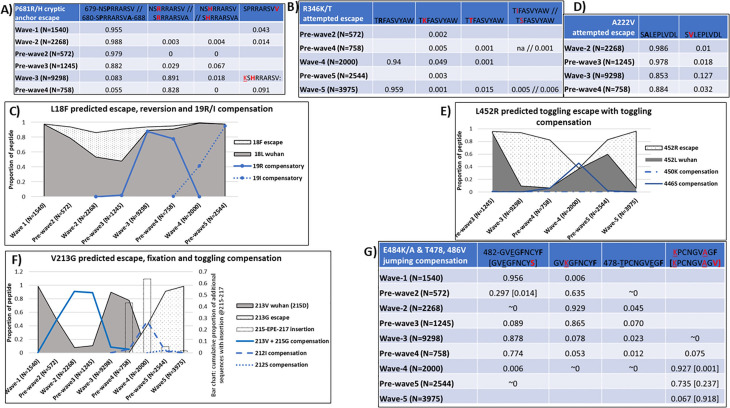
Escape patterns associated with other different HLA alleles. The proportions are shown as follows in all graphs: Wuhan residue = solid grey area; predicted escape variant = dotted area; predicted compensatory mutations = different styles of line graphs; insertions = dotted bar graphs; escaping anchor position = bold-face font residue on the peptide; compensatory position = underlined residue on the peptide. **A)** HLA-B*08:01 binds Wuhan and both P681R/H escape variants including the later N679K compensation. B*56:01 binds the overlapping Wuhan peptide and a short-lived A688V C-term escape. Peptide overlaps with the C-term of DRB5*01:01 motif and 681 is adjacent to the C-term anchor in 674-YQTQTNSP**R**-682 (15mer = 671-CASYQTQTNSP**R**RAR), variants Q675H & H677Q, T678I are also binders. **B)** Toggling of attempted escape variants driven by B*27:02 and B*27:03 which both bind to the Wuhan peptide and 346T variant. B*27:02 also binds to the 346K variant. **C)** Proportions of predicted escape, reversion and compensation at C-terminal anchor position for HLA- A*02:05 binding peptide ‘10-LVSSQCVN**L**-18’. **D)** HLA-A*02:05 and several A*02 type alleles and/or numerous C alleles drive escape but also bind escape variant due to a supermotif. **E)** Toggling between predicted escape and the Wuhan residue at C-term anchor position for the HLA- A*02:05, A*02:06, A*02:14 and A*30:01 binding peptide’444-KVGGNYNY**L**-452’. The escape variant 452R is a binder to A*30:01, A*03:01 and A*68:01. **F)** Proportions of predicted escape V213G and fixation at second anchor of 212-L**V**RDLPQGF-220 peptide binder to HLA-B*15:02 and B*07:02, with toggling of compensatory mutation sites L212I/S and D215G within the peptide. A 215-EPE-217 insertion during wave-4 is a potential escape mechanism. DQA1*01:02-DQB1*06:02 binds 215G compensatory mutation in variant 207-HTPINLVR**G**-215 (15mer = 204-YSKHTPINLVRGLPQ). **G)** Overlapping Wuhan peptides. C-term F490S mutations short-lived and binds A*24:02/A*26:01. Compensation toggling between E484K/A + T478K. Wuhan and compensations are binders to B*07:02 or B*15:08 or B*35:01. Peptide 478-TPCNGVEG**F**-486 has C-term F486V escape from B*15:08/B*35:01 motifs.

An attempted escape mutation P681R/H was observed in the B*08:01 binding peptide, 679-NSPRRARSV-687. Both Wuhan and escape variants are strong binders to the same allele (Fig 4A). A B*08:01 binding N679K compensation variant was also observed. An overlapping B*56:01/B*56:02/B*56:03 binding peptide 680-SPRRARSVA-688 had a C-term A688V escape mutation which failed early in wave-2 due to a binding supermotif (an anchor motif recognized by many HLA) shared by the B*56 HLA alleles and numerous other alleles belonging to HLA types A*26, A*30, B*15, B*58, C*03 and C*12 (S5 Table). The DRB5*01:01 allele was observed to bind Wuhan and all variants in an overlapping peptide 674-YQTQTNSPR-682 (15mer = 671-CASYQTQTNSPRRAR-685). The Wuhan peptide spanning positions 679–688 decreased in frequency over time from 98.8% in wave-2 to around 5% pre-wave-4. Although the 681H variant failed to increase, the 681R variant increased to over 80% during the same period. The selective loss of the Wuhan variant and 681H mutation was confirmed in these being directional evolution targets.

Several different escape variants were attempted at the second anchor position of HLA B*27:03/B*27:02 binding peptide 345-T**R**FASVYAW-353 (**Fig 4B**). Variant residues I/S/T/K were attempted as early as wave-2 and only R346K (4.9% in wave-4) and R346T (1.5% in wave-5) managed to increase beyond 1%. The latter two variants and the Wuhan residue were significant directional evolution targets. Both HLA alleles bind the Wuhan residue and B*27:02 also binds the 345K variant, which was present throughout all waves.

The HLA A*02:05 was associated with three escape variants. The *first* was the C-terminal anchor immune escape L18F in ’10-LVSSQCVN**L**-18’. The 18F variant emerged during wave-1 at 0.2% and increased to 42.6% during wave-2 before a sharp decline to <5% during wave-3 and nearly absent by wave-4 (**Fig 4C**). The decline was likely due to 18F being a binder to HLAs A*26:01 and B*15:02. The reversion to Wuhan L18 during wave-3 coincided with emergence and sharp increase of an adjacent T19R mutation. The 19R variant was short-lived and replaced by 19I during wave-4, while the Wuhan L18 remained above 98%. The 19R compensatory mutation was short-lived probably due to being a strong binder to HLA-B*73:01 in an overlapping ’18-L/F**R**TRTQLPP-26’ peptide with and without the 18F mutation. However, the replacing 19I variant was a binder to HLA-B*15:16. The Wuhan L18, 18F escape and the 19I compensatory mutation were confirmed as directional evolution targets.

The *second* was an attempted A222V escape mutation between wave-2 and pre-wave-4 at the second anchor position of 221-SALEPLVDL-229 peptide (Fig 4D). The A222V mutation emerged at 1.1% in wave-2, increased to 12.7% in wave-3 but quickly dropped to <3.5% before wave-4. Both A222 and 222V are found in a supermotif which binds to several HLA-A*02 alleles and numerous HLA-C alleles (S5 Table). The *third* was the toggling of the C-term anchor L452R mutation from start of wave-3 until wave-5 in the 444-KVGGNYNYL-452 peptide (Fig 4E). The Wuhan peptide is a binder to HLA A*02:05, A*02:06, A*02:14 and A*30:01. Compensation at 450K was attempted during wave-3 but failed. Another short-lived compensation appeared at G446S during wave-4 at high proportion (45%) and binds to HLA- B*15:16, B*15:17 and B*57:01 in an overlapping peptide 445-VSGNYNYLY-453. The increase of the compensation during wave-4 coincided with a decline of the predicted escape variant from 84% in wave-3 to under 1% in wave-4. However, by wave-5 the escape variant had increased rapidly to over 90% without any adjacent compensatory mutations.

The remainder two escape mutations predicted during the South Africa infection waves were associated with B*07:02. The *first* had a motif shared with B*15:02. A delayed but rapidly increasing escape V213G, at the second anchor position of the 212-L**V**RDLPQGF-220 peptide appeared after wave-3 following toggling compensation at positions 212 and 215 (**Fig 4F**). The frequency of this Wuhan peptide decreased rapidly from >98% during wave-1 peak to <10% by wave-2, coinciding with a D215G compensation variant. Although this variant increased to over 90% during wave-2, it was short-lived and complete reversion of the compensation occurred by wave-3. The 215G compensation variant is a binder to HLA-II haplotype DQA1*01:02-DQB1*06:02 in the 207-HTPINLVR**G**-215 (15mer = 204-YSKHTPINLVRGLPQ) variant peptide. This reversion was immediately followed by the V213G escape coupled with emergence of L212I compensation by wave-4. Some viruses without the V213G escape mutation emerged during the same time with a 215-EPE-217 insertion. The 212I compensation nearly occupied 50% of circulating strains but disappeared by end of wave-4 coinciding with emergence of 212S which remained <5%. All sequences with the 212I mutation had a 211N deletion. The V213G escape dominated to >98% during wave-5 without any major compensation within the peptide. The proportion of the 215-EPE-217 insertion in the combined sequence data was 43.1%, 63.8%, 5.5% and 0.2% in pre-wave-4, wave-4, pre-wave-5 and wave-5, respectively. No motif was found in peptides with insertions. Wuhan residues in all compensatory and escape positions and the 215G variant were the directional evolution targets.

The *second* had a motif shared with HLA- B*15:08 and B*35:01 and associated with a short-lived F490S C-term mutation in the 478-TPCNGVEG**F**-486 peptide, at 1.4% after the wave-1 but disappeared before the next infection wave peak. This mutation coincided with emergence of E484K compensatory mutation in viruses without the 490S mutation, which increased to >90% during wave-2 but was completely replaced by 484A during wave-4 (**Fig 4G**). The 490S mutation enabled binding to HLA- A*24:02 and A*26:01. The emergence of 484A appeared to coincide with another compensation at T478K and a new rapidly increasing C-term anchor F486V mutation in the same sequences reaching >90% by wave-5. The immune escape and all compensatory positions were directional evolution targets.

### Other predicted zoonosis related immune escape patterns

**Fig 5:**
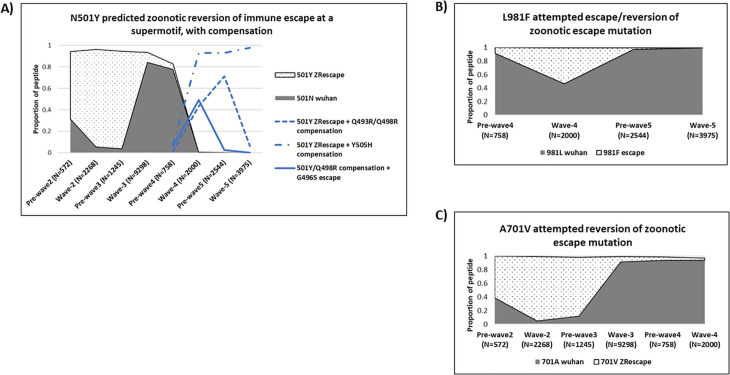
Zoonosis related escape patterns associated with other different HLA alleles. The proportions are shown as follows in all graphs: Wuhan residue = solid grey area; predicted escape variant = dotted area; predicted compensatory mutations = different styles of line graphs; **A)** N501Y predicted reversion of zoonotic immune escape at C-term anchor position of a supermotif in the HLA- B*15:16/ B*15:17/ B*57:01/ B*58:01/A*03:01 binding variant peptide ‘493-QSYGFQPT**Y**-501’. The mutation has toggling compensation while it also acts as a compensation for an overlapping peptide 495-YGFQPTNGV-503. DRB1*01:01 binds N501 and the variant 501Y, escape is at the compensatory positions 496S and 498R, 498 is an anchor in 495-YGFQPTNGV-503 (15mer = 492-LQSYGF**Q**PTNGVGYQ). **B)** L981F attempted escape mutation or reversion of zoonotic escape mutation at C-term of 973-ISSVLNDIL-981 peptide. The variant is a strong binder to B*58:01/B*57:02. The Wuhan peptide was predicted as a weak binder to same alleles but in absence of known anchor residue motif. DRB1*11:04 also binds same Wuhan and variant on the N-term anchor in 981-LSRLDKVEA-989 (15mer = 978-NDILSRLDKVEAEVQ). **C)** A701V predicted attempted reversion of a zoonotic immune escape at C-term anchor position of 693-IAYTMSLGV-701 variant peptide with a strong binding motif for HLA- B*51:01/ B*51:02/ B*51:03 alleles. The Wuhan peptide is not a binder to any HLA. DRB1*07:01 binds both Wuhan (weak) and variant (strong) in C-term anchor of 693-IAYTMSLG**A** -701 (15mer = 690-QSIIAYTMSLGAENS).

The B*58:01, along with other several HLA alleles was associated with two of the six predicted zoonotic escape mutations. The *first*, N501Y mutation is at the C-term anchor position of a super-motif in 493-QSYGFQPTN-501, where the 501Y variant binds to HLA- B*15:16/ B*15:17/ B*57:01/ B*58:01/A*03:01 while the Wuhan N residue isn’t a binder to any of these alleles. However, HLA- DRB1*01:01 binds to both N501 and 501Y. Although the variant peptide with only the N501Y mutation increased during wave-2, it decreased thereafter to <1% by wave-4 at which a multi-variant peptide with N501Y, Q493R and Q498R mutations emerged to >40% frequency (Fig 5A). The multivariant peptide increased to 71.1% pre-wave-5 but dropped rapidly to 6.4% during wave-5. The changes during wave-4 to wave-5 coincided with emergence and rapid increase to 97.9% of an overlapping peptide containing a non-anchor Y505H mutation and N501Y as a second position anchor mutation observed to bind to A*30:02, A*30:03 and several HLA C alleles (S5 Table). In addition, the Y505H mutation was a predicted second and C-term position anchor for other HLA alleles when 501Y was present at a non-anchor position. N501Y mutation was also presented as a non-anchor position in the overlapping peptide 495-YGFQPTNGV-503 where co-existing mutations were observed at second anchor G496S for HLA- B*15:16, B*51:01, B*51:02, B*51:03. The G496S mutation was short-lived during wave-4 while the peptide variant with N501Y and Q498R mutations emerged around wave-4 and rapidly increased to 98.5% by wave-5. The zoonotic directional evolution targets were: 501Y, 493R, 498R and Wuhan residues for 496, 498 and 505.

The *second* B*58:01 associated zoonotic mutation was at position L981F, in the Wuhan peptide 973-ISSVLNDI**L**-981. The variant introduced a strong binding anchor motif for HLA- B*58:01 and B*57:02. The variant was in >50% of circulating sequences during wave-4 but was short-lived to <1% by wave-5 (**Fig 5B**). The Wuhan peptide does not have the known motif for these alleles but is a binder. Both the Wuhan and variant peptides bind to the HLA-II allele DRB1*11:04 at the N-term anchor in 981-LSRLDKVEA-989 (15mer = 978-NDILSRLDKVEAEVQ). The Wuhan L981 was the target for zoonotic directional evolution.

The sixth predicted zoonotic mutation was A701V variant in the C-term anchor of 693-IAYTMSLG**A**-701 peptide which appeared at high frequency (61.4%) after wave-1 and increased rapidly to >90% by wave-2 (**Fig 5C**). It rapidly decreased thereafter allowing the Wuhan peptide to occupy over 94% of circulating sequences. The variant contains a binding motif for HLA- B*51:01, B*51:02, B*51:03, A*02:14 and A*69:01 alleles while the Wuhan peptide lacks the motif and is not a binder. Both the Wuhan and variant peptides were targets for zoonotic directional evolution and binders to the HLA- DRB1*07:01 in the 15mer peptide 690-QSIIAYTMSLG**A**ENS-704.

## Discussion

Even though the importance of T cell immune responses in controlling SARS-CoV-2 infection independent of neutralizing antibodies has been confirmed [42–45], there is limited research to understand the direct effect of the T cell immune response pathway on the emergence and persistence of SARS-CoV-2 immune escape variants. We hypothesized that surveillance of amino acid anchor motifs preferred by the highly restrictive peptide binding grooves of HLA molecules can highlight population level footprints of the most important SARS-CoV-2 adaptative T cell immune escape variants associated with infection spread. Given the high polymorphism of HLA alleles and hence varying frequencies between ethnic populations plus the observed geographic variations in timing of emerging SARS-CoV-2 lineages and variants, we analyzed HLA associated escape in SARS-CoV-2 viruses circulating within a defined geographic sub-region. Even though other Bioinformatics predictions of T cell targets in SARS-CoV-2 proteins have been useful in listing globally dominant T cell targets [16–18,46], this study has added a new approach to shortlist immune-dominant epitopes which are susceptible to immune escape. The proposed method strengthens the specificity of the prediction of escape mutations by focusing on the highest affinity binding anchor positions in SARS-CoV-2 data isolated from a defined geographic population and confirmed by phylogenetic evolutionary analysis. The results of this study largely confirmed our hypothesis: a streamlined list of 17 peptides with predicted immune escape at HLA targeted anchor motifs, was produced from SARS-CoV-2 Spike protein sequence data isolated in South Africa from the start of the COVID-19 pandemic through to June 2022. These peptides were successfully predicted to be involved in dominating (i.e., same peptide observed to have anchor variants during multiple infection waves) T cell immune responses against SARS-CoV-2 during the first five COVID-19 infection wave peaks in South Africa. Characteristic toggling of short-lived escape and compensatory mutations was observed and explained by overlapping HLA binding preferences and known underlying antibody pressure and structural constraints. All peptides were linked to escape at HLA-I anchor motifs, eight also overlapped with escape at HLA-II anchor motifs and six had escape mutations predicted to be associated with zoonotic adaptation. The HLA- A*66:01/A*68:01 alleles were associated with nearly half of the immune escape peptides. Other HLA alleles associated with at least two escape peptides individually or with other alleles sharing the same anchor motif were HLA-I A*02:05, A*03:01, B*07:02, B*08:01, B*58:01, and HLA-II DRB1*04:01 and DQA1*01:02-DQB1*06:02. All predicted immune escape sites were under significant directional evolution on the phylogenetic tree.

*Analyzing viral data isolated from a* ***defined geographic region exposed the overlooked influence of HLA polymorphism on the T cell immune response against SARS-CoV-2 in understudied populations***. Previous systematic reviews of several (N ≥ 18) studies across the globe reporting many unique SARS-CoV-2 T cell epitopes, identified HLA A*02:01 to be overly dominating the T cell response repertoire, followed by A*24:02, A*01:01 and B*07:02 [47,48], yet escape associated with A*02:01 was not identified in any dominant immune escape peptide in this data from South Africa. Only the B*07:02 allele identified in our findings is listed among the globally common T cell immune responses against SARS-CoV-2. In the published review, A*68:01 was associated with half the number of T cell epitopes as A*02:01 yet the SARS-CoV-2 Spike sequences circulating during the South Africa infection waves appeared to be enriched with A*66:01 and A*68:01 anchor motifs undergoing selective pressure [40,47–49]. The HLA-A*68:01 allele is present in all ethnic groups in South Africa at frequencies ranging between 2% an 8%, and at a proportion of >0.16 as a A*68:01 ~ DQB1*02:01 haplotype, strongly supporting this study’s hypothesis and the need to investigate virus escape patterns in relation to local population immune response genotypes [40,50]. The HLA- A*03:01 had the same ranking as A*68:01, while A*08:01 and DRB1*04:01 were associated with fewer epitopes in the global studies [47]. Even though published studies focused on globally immune-prevalent and conserved peptides, none of the other alleles nor any peptides dominant in this study were highlighted there. The bias towards globally common alleles and highly conserved epitopes excludes immunodominant but not highly conserved epitopes and regionally dominant immune responses, which should be considered in broadening immunogen coverage of globally effective vaccines. In this dataset, toggling of escape and compensatory mutations was the most dominant feature, reflecting the nature of HLA polymorphisms in the ethnically diverse population of South Africa.

*E****vidence of overlapping HLA-I and HLA-II immune response pressure*** *was observed from the predicted patterns of* ***toggling or delayed immune escape****.* Three of the predicted HLA-A*68:01 associated peptides overlapped with HLA-II predicted immune pressure. In the T95I mutation associated with A*68:01 and DRB1*04:01 immune responses, toggling was likely due to A*68:01 which also recognized the emerged variant even though the residue is absent from the known anchor motif. These binding predictions are supported by previous studies, where the 15-mer peptide was a ligand to DRB1*04:01 while an overlapping 9-mer (positions 89–97) showed T cell activity against samples from A*68:01 positive convalescent donors [51,52]. The toggling was supported by directional evolution, where both residues were targets on the phylogeny. The delayed A*66:01/A*68:01 R408S escape mutation with the D405N compensation was predicted to bind HLA-I DQA1*01:02-DQB1*06:02 allele, most likely explaining its delayed emergence and need for compensation. The pre-existence of HLA-II pressure was supported by significant directional evolution towards the Wuhan residues in both the escape and compensatory positions. The emerged variants were not significant evolutionary targets, likely indicating the dominance of CD4 T cell immune pressure over CD8. HLA- A*68:01 recognition of the same peptide is supported elsewhere where activated T cell immune response was observed in convalescent samples of a A*68:01 positive individual [52]. The Wuhan G769 residue was a predicted zoonotic escape mutation driven by a supermotif containing the sporadic variant 769V which binds HLA-I alleles A*66:01/A*68:01, A*02:06, A*02:14, and HLA-II DQA1*01:02-DQB1*06:02 haplotype. The peptide at 764–780 has been observed to elicit CD4 T cell immune responses in convalescent donor samples of DQA1*01:02-DQB1*06:02 positive individuals [52,53]. The Wuhan G769 residue was a significant zoonotic directional evolution target, supporting the predicted pressure on the supermotif posed by the variant 769V. The failure of the G769 escape to revert to V, appears to have been compensated by an overlapping HLA-II DRB1*04:01 selective pressure leading to a N764K mutation. The Wuhan N764 was a predicted N-terminal anchor for DRB1*04:01. Indeed, the N764K mutation has previously been attributed to the DRB1*04:01 allele in transgenic mice studies and further supported by binding of DR4 alleles to the same peptide region in yeast display assays [51,54].

Two other predicted HLA-I zoonotic mutations N501Y and A701V overlapped with HLA-II binding peptides. The HLA- B*58:01 predicted N501Y mutation is located in an immunogenic region and known to evade neutralizing antibody responses, thus further supporting the possibility of a zoonotic footprint [55]. The 501Y variant introduced a supermotif for several B*58 and B*15 alleles while the Wuhan N501 was a non-binder escape mutation, supporting the observed rapid decrease of the 501Y variant during the early infection waves. The predicted toggling compensatory mutations (493R, 496S & 498R, 505H) within this peptide immediately following reversion of N501Y mutation, could be in part due to the predicted binding of HLA-II DRB1*01:01 to both N501 and 501Y or overlapping antibody selective escape previously observed at positions 496 and 498 in the receptor binding domain [56]. The 505H mutation has previously been reported to be important for virus protein function although the mechanisms are not yet clear [57]. The zoonotic selective force towards the 501Y variants was evident on the SARS-CoV-2/sarbecovirus phylogenetic tree. Significant directional evolution was also evident in all predicted compensatory mutations, affirming the extensive selective forces in this peptide region.

In the case of A701V, DRB1*07:01 binds to both the A701 peptide and the 701V variant peptide. Presentation of the A701 peptide by the same allele has been previously reported in mass spectrometry assays [58], probably explaining the high increase in the proportion of the emerged variant during wave-2. However, the rapid decline of the emerged variant to a sustained extremely low frequency after infection wave-2 could be explained by binding to the variant by several HLA-I alleles B*51:01, B*51:02, B*51:03, A*02:14 and A*69:01 or an underlying functional constrained for its proximity to the furin cleavage site.

HLA-I escape was also observed to overlap with E484, another known neutralizing antibody target on the receptor-binding domain, that is susceptible to immune escape commonly through 484K, with the ‘A’ escape occurring after prolonged infection [55,56,59–61]. In this study, E484K/A was a predicted compensatory position for HLA-I escape mutations. Strong early immune escape at C-term peptide F490S associated with the A*24:02 and A*26:01 alleles toggled with reversion coinciding with several compensatory mutations, including jumping toggling at E484K/A compensatory position. The latter appeared to enable successful overlapping B*07:02, B*35:01, B*35:05 and B*53:01 F486V escape (or compensation for F490S). All predicted escape and compensatory residues in the latter case were significant directional evolution targets, indicating the strength of the B*07:02/B*35:01/B*35:05/B*53:01 immune pressure on F486. Even though co-evolution analysis was not conducted in this study, the dependency between the escape position and compensatory mutations was evident.

HLA- B*08:01 predicted immune escape also overlap with HLA-II binding peptides. The predicted delayed but sharp increase of the P681R mutation (to over 80% during wave-3 onwards) is likely due to a combination of B*08:01 and DRB5*01:01 alleles which bind to both Wuhan and emerged variant residues. The delay in fixation of 681R was confirmed by a significant directional evolution pressure towards the Wuhan residue and a short-lived alternate 681H mutation. The association of this peptide with B*08:01, has been reported elsewhere in T cell assay activity of convalescent samples from individuals bearing this HLA [52]. Another allele, B*56:01, was a predicted strong binder to the Wuhan P681 likely contributing to the selection of 681R in the later infection waves. Laboratory assays may be required to confirm the contribution of B*56:01 and DRB5*01:01.

***Toggling or delayed immune escape was explained by densely overlapping HLA-I anchor motifs and/or other underlying competing selective pressure.*** The same peptide with P681R in the second anchor position had a C-term A688V mutation located within a supermotif. The Wuhan peptide was associated with HLA- B*56:01/B*56:02/B*56:03 and overlapped with a B*08:01 cryptic motif. The 688V variant was short-lived and likely because it introduced a supermotif which binds numerous alleles including those belonging to types A*26, A*30, B*15, B*58, C*03 and C*12 (S5 Table). Immune responses associated with A*26:01 and B*57:01 against the same peptide region have been reported in a study of convalescent samples [52].

The predicted HLA-A*66:01 and/or A*03:01 driven toggling escape at S371L/F was due to HLA- B*44:02 and B*44:03 binding the emerged 371L/F variants in an overlapping C-term anchor. The high selective pressure exerted on this site appeared to be associated with ***jumping toggling compensation*** within the same epitope, also influenced by complex overlap of other potential HLA anchor binders. Compensatory mutation 375F (but not Wuhan 375S) was a C-term binder to B*15:02 and could be the reason for the additional compensation by T376A mutation which sustained the S371F escape variant to increase to >90% towards infection wave-5. The coinciding S373P mutation could have emerged not primarily to compensate for S371F escape but likely an escape mutation from second position anchor binding in what appeared to be a supermotif in 372-ASFSTFKCY-380 for several HLA alleles including B*15:02, B*15:16, B*57:01 and C*07:03 (S5 Table). These positions are also under functional constraints of the ACE-2 receptor binding domain (RBD) and the observed mutations have been indicated to reduce virus infectivity fitness [62].

The A222V and L452R mutations associated with A*02:05 allele also appeared to occur within peptides containing a dense overlap of anchor motifs. In L452R, located within the RBD and a neutralizing antibody target, several HLA alleles (including HLA- A*02:05, A*02:06, A*02:14 and A*30:01) bind to a common ‘L’ anchor at the C-terminus, but the escape failed, likely due to the functional requirement of the RBD [56,63]. The variant 452R is also a weak binder to HLA- A*30:01, A*03:01 and A*68:01. A predicted compensation at G446S was short-lived possibly due to a combination of the functional requirement as an ACE-2 receptor attachment site and potential binder to several alleles in an overlapping peptide (S5 Table) [63]. The L452R mutation has previously been observed in an individual with prolonged COVID-19 disease [64]. The A222V mutation is also located within the N-terminal domain region and notable for its importance in infectivity and transmissibility fitness [65,66]. Interestingly for the A222V mutation, the ‘A’ residue is not in the published motif but forms a strong binder to A*02:05 and A*02:06 and a weak binder to A*68:02 (S5 Table). Similarly, the variant ‘V’ is a predicted binder to several C alleles yet absent from the known anchor motifs required by these alleles (S5 Table). The mismatch between binding and the known anchor motifs indicate potential novel anchor residues in SARS-CoV-2 which need to be confirmed *in-vitro*.

The attempted and failed R346T/K escape variants were associated with HLA- B*27:02 and B*27:03 which bind to both the Wuhan peptide and at least one of the emerged variant residues. The toggling variants, also significant directional evolution targets, remained at low frequency (<5%) implying other underlying suppressive pressure. This site is located in the RBD and the mutations have been indicated to support transmissibility and evasion of antibody immune responses [67,68].

The zoonotic footprint in position 440 would be expected given it is in the highly variable receptor-binding motif targeted by several monoclonal antibodies [69]. The N440K mutation has previously been observed in an individual with prolonged COVID-19 disease [64]. Although not observed occurring with N440K in this study, the adjacent N439K mutation has been reported to increase spike affinity to the human ACE-2 receptor while evading antibody immune response [69]. It still needs to be investigated whether these two positions co-function.

***Insertions and deletions are potential immune escape mechanisms for sites with densely overlapping selective pressure.*** Complex toggling of predicted compensatory mutations within a B*15:02 and B*07:02 motif-containing peptide ‘212-LVRDLPQGF-220’ was observed between D215G, L212I and the 215-EPE-217 insertion. Interestingly, these toggling compensations preceded a delayed B*15:02/B*07:02 anchor V213G escape mutation. The D215G mutation was the first attempted compensation but was short-lived possibly due to the 215G introducing a binding C-term anchor to DQA1*01:02-DQB1*06:02 in an overlapping peptide. The reversion to D215 coinciding with the emergence of L212I mutation and 215-EPE-217 insertion and subsequently the delayed B*15:02/B*07:02 V213G escape mutation at the start of infection wave-4. It is interesting that none of the compensations were present during wave-5 as the 213G escape showed fixation. The L212I compensatory mutation resulted in strong binding affinity to many alleles belonging to types A*26, B*15, B*58 and B*35, possibly explaining why it was short-lived. A 211N deletion was observed in all sequences with the L212I mutation, a possible co-compensation. The 215-EPE-217 insertion which persisted throughout wave-4 is also a potential alternative to the failed D215G compensation. No motif was found in this insertion variant peptide making it a potential immune escape mechanism from HLA-B*15:02/ B*07:02. The fixation of the delayed V213G escape mutation, after losing these numerous compensations could reflect the strength of the HLA-I B*15:02 and B*07:02 immune pressure in this region during later infection waves. Indeed, of the HLA alleles which bind the peptide 212-LVRDLPQGF-220 exhibiting this toggling, HLA-B*15:02 is present in the South African Indian population at 3% and B*07:02 at 8% in the South African population [40,70]. It is also possible that stronger compensation was later achieved outside of the peptide. Alternatively, there could have been dominating overlapping neutralizing antibody immune response pressure during the earlier infection waves which prevented the V213G mutation (unique to the BA.2 omicron sub-lineage), that later waned widely at population level. The role of DQA1*01:02-DQB1*06:02 needs to be confirmed. The prediction of HLA- B*07:02 pressure in this peptide region as well as inference of diminished overlapping HLA-II responses supports similar findings previously reported by Nersisyan et al, who observed a decline in HLA binding affinity due to the N211 deletion, L212I and V213 mutations and the ‘EPE’ insertion [71]. The dominance of HLA- B*07:02 T cell responses in disease control has also been demonstrated in transgenic mice studies [72].

The ***role of frequent HLA alleles*** contributed to toggling of escape and compensatory variants. The A*66:01 and A*68:01 alleles are typical examples. Both alleles are present in all major ethnic groups in South Africa [40]. The predicted attempted reversion of a short-lived zoonotic escape N856K was explained by the binding of A*66:01/A*68:01 and B*08:01 alleles to the emerged mutation. An overlapping peptide, previously reported to have strong presentation by HLA-II DRB1*15:01 could have contributed to the toggling, even though it lacked a published anchor motif [58]. The toggling of the predicted C-term anchor escape K417N in the A*03:01/A*66:01 binding peptide is expected given that K417 is one of the ACE-2 receptor attachment positions in the RBD [69]. However, the 417N variant originated within South Africa without notable presence globally and this can be attributed to the high frequency of HLA A*66:01 in this population [73].

In the zoonotic mutation L981F, the 981F mutation was short-lived even though the dominating Wuhan L981 is a strong binder to HLA-II DRB1*11:04. Another study reported the 981F mutation inducing increased infectivity fitness [62]. It is possible that 981F was short-lived due to binding of B*58:01 which is present in all major ethic groups in South Africa at a frequency range 0.02-0.07 [40].

The immediate L18F immune escape during wave-1 is expected when considering that the HLA-A*02:05 has a frequency of 6% in the Black-African ethnic group in South Africa [40]. This association is also supported by that this variant was first observed and contained in only South Africa and Brazil [73]. The HLA which recognize the escape variant 18F have been reported to be found in 1–2% (A*26:01 in all main ethnic groups) and 1–3% (B*15:02 in non-Black African main ethnic groups) of the South African population. Either these or other underlying fitness costs, or a combination of both pressures, forced the need for position 19 compensatory mutation coinciding with early reversion at position 18, during the end of wave-2. The observed short-lived compensation for L18F reversion during the end of wave-2 by 19R and its replacement by 19I which increased to dominance could also be related to HLA allele frequency in the local populations. HLA-B*15:16 which binds to 19I at the C-terminal anchor position has a comparatively low frequency in the different ethnic groups in South Africa, ranging from 0.5% in Caucasians to 1.4% in Black-Africans [40]. The frequency of HLA-B*73:01 which binds strongly to 19R as a second anchor is unclear in South Africa but found at very low frequency in other southern/eastern Africa ethnic groups, 0.2% in Luo/Nandi (Kenya) and Shona (Zimbabwe) [40]. About 5% of sequence data, also bearing the 19I mutation in the C-terminus position had a 24-LPP-26 deletion. The 19I mutation, along with position 18 escape and Wuhan variants were significant evolutionary targets on the phylogeny. Positions 18 and 19 are also located in the exposed surface of the N-terminal domain, forming an antibody target supersite, in addition to the competing functional pressure [58,65,73].

***Importance of compensatory mutations was evident.*** Escape mutations in nine of the seventeen peptides were predicted to correlate with compensatory mutations. The abundance of compensatory mutations and toggling of these to some extent revealed differences in the strength of immune response pressure exerted on different amino acid positions. Future work using phylogenetic models for identifying co-evolving codon positions would be useful in confirming linkage between escape and compensatory positions.

***HLA-induced toggling of short-lived escape is an inter-host population level characteristic*** Combining our immunoinformatic prediction of HLA associated escape and what has been observed about T cell immune response against SARS-CoV-2, we hypothesize that the observed toggling of T cell escape is largely a result of inter-host than intra-host selective pressure. The very large dataset used here has no information on donor characteristics but was isolated from different types of donors across South Africa, i.e., healthy, immune-compromised, unvaccinated, vaccinated, primary infections and possibly re-infections, but likely dominated by primary unvaccinated infections (sequenced from the start of the pandemic until mid-2022). Our results therefore give a representative population-level snapshot of the most dominant HLA-associated evolutionary patterns in the local population. In addition to the fact that neutralizing antibody immune response precedes T cell immune response during primary infection, other studies across the globe have indicated: preservation of T cell immune responses among vaccinees, particularly during the first year post vaccination [74]; that T cell immune response is active in controlling COVID-19 disease progression, showing minimal activation in healthy individuals yet highly activated in severe disease and only showing escape after prolonged infection [64,75,76], and that primary (infection or vaccine-induced) neutralizing antibody immune response against SARS-CoV-2, even though has an infectivity protective capacity tends to wane over time [77–79]. At individual host level, when the T cell escape mutations are transmitted, reversion is highly likely to occur during acute infection due to the collateral damage from overlapping antibody epitopes and/or to subsequent mismatched HLA genotypes in the recipient host. The latter is indeed expected in SARS-CoV-2 given the observed large breadth and diversity of T cell targeted peptides between individuals due to HLA polymorphism [52]. Thus, the overlap with antibody epitopes combined with HLA-mismatched recipient hosts in this genetically diverse population and commonly shorter or even absence of chronic infection (which otherwise would allow fixation of T cell escape mutations) in many individuals hinders fixation of HLA induced escape after transmission. These overlapping effects combined with varying levels of CD8 T cell activation, wherein, the majority of the population exhibits low to medium activation levels [80–84], result in what surfaces as toggling of attempted/failed/short-lived escape at population level.

***Considerations for vaccine design.*** We produced a list of HLA-associated peptides with anchor mutations observed in at least two non-adjacent infection waves/inter-waves, a simplified indication of potential population-level immunodominance, and worth considering for vaccine immunogens. However, most of the identified HLA alleles are not listed in globally dominant T cell immune responses, which also focused only in highly conserved epitopes [16,46,74]. Unfortunately, this potentially excludes immunodominant but ethnically localized T cell immune responses as well as settings where epitope sequence conservations may be limited due to exposure to highly diverse immune genotype profiles of the host population. Our results raise a question of whether including immunodominant peptides with mostly short-lived escape mutations together with globally dominant conserved epitopes, in vaccine immunogens, could broaden vaccine effectiveness. In a population with high HLA polymorphism, would toggling of short-lived escape in immunodominant peptides of a vaccine outweigh vaccine effectiveness? Including such immunogens might not increase the breadth of efficacious responses at individual intra-host level but could improve breadth of responses at population level, compared to immunogens containing only highly conserved epitopes, given that conserved immunogenic peptides tend to be for select HLA genotypes [52,74,75]. Understanding the fate (short-lived or fixation) of T cell escape mutations in long-term infection individuals, immunocompromised persons and those with severe disease is also important in the consideration of using partially conserved vaccine immunogens.

### Limitations

The method developed here focusses on identifying mutations which evade only one step of the T cell response pathway, the HLA-binding step. Although this step may fail to identify epitopes whose adaptive mutations are related to other steps on the T cell immune response pathway, it is on its own a strong predictor of failure of an immune response. The HLA antigens further make it possible to relate the results directly to the host populations and increase our understanding of the general pathogenesis profile within a defined population.

Only 9-mer and 15-mer HLA-I and HLA-II peptipe binders were investigated, respectively and future work will explore other peptide lengths. The data were further restricted by availability of HLA binding information in the binding affinity prediction software, thus some important escape mutations are likely to have been missed. The association of mutations with some alleles and not others could also be a coincidence of over-representation of anchor motifs for these alleles in SARS-CoV-2. The sequence data used were not linked to the donor’ specific ethnicity nor HLA genotype. However, it is impossible to obtain such large sequence dataset all matched to host HLA. The big sequence data analysis outweighs this limitation and provided important results for guiding streamlined *in vitro* studies of immune escape in addition to revealing the impact of HLA polymophism in the ethnically diverse population of South Africa.

### Unique strengths

Several immunoinformatic predictions of SARS-CoV-2 T cell epitopes have been conducted since the beginning of the pandemic, but prediction of escape mutations has been scarce. Given that single point mutations have previously been confirmed to enable CD8 T cell evasion in SARS-CoV-2 [85], we produced a method which prioritizes the highest binding affinity anchor positions on HLA-binding peptides, to identify the most important escape mutations. This approach also provides a first step towards distinguishing between escape mutations and compensatory positions. Thus, we successfully produced a streamlined list of non-conserved immunogenic peptides for monitoring and contribute to prioritization of laboratory T cell epitope and immune escape validation experiments. Already, all but one (A222V known for infectivity and transmissibility fitness) immune escape sites predicted in this study (including some predicted as compensations), L18F, T95I, V213G (and 215-EPE-217 insertion), S371F/L (and S373P, S375F), R346K/T, R408S, K417N, N440K, L452R, E484K, N501Y (and Q493R, Q498R), P681R/H (and A688V), A701V, G769V, N856K and L981F, have been confirmed in laboratory assays or identified in silico, to be either within epitopes or immune evasion mechanisms associated with CD8 T cell response or HLA-I binding (although some for different commonly studied HLA), S5 Table [43,51–53,64,71,75,86].

The observed underlying noise of attempted T cell escape enlightens our understanding of the determinants of breadth of vaccine-induced immune responses in this southern Africa region.

The other novelty is the use of a phylogenetic model for directional evolution to affirm the predictive power of the immunoinformatic approach used. The phylogenetic model further confirmed the most dominant selective force either toward an escape variant, reversion, or both. Molecular evolution models, although not focusing on directional evolution, have been used to successfully identify important point mutations in SARS-CoV-2 genetic sequences [87]. In our approach, we were also able to predict point mutations which likely enabled the virus to adapt to the human host during zoonosis. The general dominance of A*68:01 in zoonosis associated escape is not unexpected as it was similarly identified for chimp-to-human HIV transmission [15].

Overall, this method opens opportunities to predict geographic spread of immune evasion, and the balance between immune escape and immunogenicity.

## Conclusion

We have developed a novel immunoinformatic pipeline supported by phylogenetic evolution, to successfully predict a streamlined list of SARS-CoV-2 immunogenic epitopes which are subject to T cell immune escape mutations associated with binding of HLA genotypes. This is one of limited, if not the first, immunoinformatic prediction of HLA-associated immune escape mutations at the high affinity binding anchor positions. Characteristic toggling of short-lived immune escape mutations was common and frequently coincided with unstable compensatory mutations. This characteristic is attributed primarily to exposure of SARS-CoV-2 viruses to high HLA polymorphisms in the ethnically diverse population of South Africa. Other observed contributing factors were the overlap between HLA-I and HLA-II recognition, underlying neutralizing antibody targets and in some cases, underlying functional constraints. The findings confirm population level cumulative ‘escape knock-out’ dynamics occurring at intra-host levels, between the early timing of strong and subsequently waned antibody immunity versus late and varying levels of activated T cell immune response as well as mis-matched HLA genotypes upon transmission of variants. By analyzing data from a defined population region, we have shown that some of the important immune responses in populations with high immunogenetic diversity, may be different from the globally common HLA and the target epitopes may not be highly conserved. Whether the toggling of these short-lived escape variants negatively impacts on vaccine efficacy or are primed at all, should these immunodominant peptides be used as vaccine immunogens, particularly during the period of waned antibody immune responses, needs to be investigated. In addition to understanding the behavior of dominant T cell epitopes in a defined geographic setting with an ethnically diverse population, we were able to reveal signals of zoonotic adaptation and highlighted the active role of compensatory mutations, both requiring future confirmatory studies.

This streamlined immunoinformatic prediction of T-cell associated selective pressure at HLA anchor motifs has high predictive power (supported by 16/17 predictions in previously reported T cell targets) and a potential to cost-effectively guide pre-clinical research for vaccine design. Overall, analyzing SARS-CoV-2 viruses isolated from an ethnically diverse population, (i)shortlisted potentially understudied population-specific HLA and immune escape (ii)revealed a footprint of underlying toggling of short-lived immune escape mutations, and (iii)questions whether including locally dominant/common T cell immune responses with toggling escape, in vaccine immunogens would increase vaccine effectiveness/efficacy for ethnically diverse populations.

## Supporting information

S1 Table Summary of the final Spike protein sequence data used. Data were isolated in South Africa from beginning of pandemic through to June 2022. Dates for different infection waves were guided by the detailed infection waves observed in South Africa published by Jassat et al (2021), Madhi et al (2022) and https://ourworldindata.org/coronavirus/country/south-africa?country=~ZAF [24,25]. All genome sequences and associated metadata that were used in this analysis are published in GISAID’s EpiCoV database. Information for the specific dataset downloaded and used for this study, including sequence accession number, Virus name, Collection date, Originating Lab and Submitting Lab and the list of Authors, can be found at https://doi.org/10.55876/gis8.240722me (identifier: EPI_SET_240722me).
(DOCX)

S2 Table GenBank Accession numbers for sarbecovirses used as a proxy for SARS-CoV-2 ancestral strains. Data were taken from an online source found at: Visualizing selection analysis results for evolution of nCOV (Nov 2021 update)/ Sergei Pond/ Observable (observablehq.com) and produced by Lytras et al (2022) [27].
(DOCX)

S3 Table HLA-I and corresponding 9-mer anchor residue motifs used (N = 137 unique HLA-motif pairs). The data was downloaded on 25 March 2022 from https://www.hiv.lanl.gov/content/immunology/motif_scan/motif_help.html#Motif_Scan_Help Four-digit HLA class I alleles with 9-mer anchor motifs containing at least two defined anchor positions were shortlisted (N = 137) and used to search for potential HLA binding peptides on the SARS-CoV-2 protein sequences.
(DOCX)

S4 Table HLA-II and corresponding anchor residue motifs used (N = 45 unique HLA-motif pairs). The data were downloaded on 25 March 2022 from https://www.hiv.lanl.gov/content/immunology/motif_scan/motif_help.html#Motif_Scan_Help. HLA class II alleles with anchor motifs containing at least two defined anchor positions were shortlisted (N = 45) and used to search for potential HLA binding peptides on the SARS-CoV-2 protein sequences.
(DOCX)

S5 Table List of escape mutations and related epitope sequences, binding affinity results and supporting evidence from other published sources. Boldface font = anchor residue Wuhan (wildtype); Red font = emerged variants (non-wuhan); Underlined residues = compensatory mutations; WB = Weak Binder; SB = Strong Binder; NTD - N-terminal domain; RBD - Receptor-binding domain; nAb - neutralizing antibody.
(XLSX)

S1 Code Python code to find amino acid positions with matched anchor residue motifs and probable escape variants associated with HLA-Class-I binding. (PY)

S2 Code Python code to find amino acid positions with matched anchor residue motifs and probable escape variants associated with HLA-Class-II binding. (PY)

S1 Text Detailed methods for Phylogenetic inference of directionally evolving sites. (DOCX)
