# Supplementary material for: Population-level toggling of T cell immune escape at human leukocyte antigen anchor residues in SARS-CoV-2 Spike proteins, in an ethnically diverse population region
Source: PLoS Comput Biol. 2025 Jul 21;21(7):e1013261. doi: 10.1371/journal.pcbi.1013261 (PMC12303384; doi:10.1371/journal.pcbi.1013261)
Supplement: S1 Table — Data were isolated in South Africa from beginning of pandemic through to June 2022. Dates for different infection waves were guided by the detailed infection waves observed in South Africa published by Jassat et al (2021), Madhi et al (2022) and https://ourworldindata.org/coronavirus/country/south-africa?country=~ZAF [24,25]. All genome sequences and associated metadata that were used in this analysis are published in GISAID’s EpiCoV database. Information for the specific dataset downloaded and used for this study, including sequence accession number, Virus name, Collection date, Originating Lab and Submitting Lab and the list of Authors, can be found at https://doi.org/10.55876/gis8.240722me (identifier: EPI_SET_240722me). (DOCX) [file pcbi.1013261.s001.docx]

**S1 Table.** ***Summary of the final Spike protein sequence data used.***

| **COVID-19 pandemic period in South Africa** | **Dates of pandemic period** | **Number of sequences (no amino acid insertions)** | **Number of sequences WITH insertions [at Wuhan-1 sites]** |
| --- | --- | --- | --- |
| Pre-Wave1 | until 30 May 2020 | 406 |  |
| Wave-1 peak | 31 May 2020 - 29 Aug 2020 | 1539 |  |
| Pre-Wave2 | 30 Aug 2020 - 28 Nov 2020 | 571 |  |
| Wave-2 peak | 29 Nov 2020 - 6 Feb 2021 | 2267 |  |
| Pre-Wave 3 | 7 Feb 2021 - 15 May 2021 | 1244 |  |
| Wave-3 peak | 16 May 2021 - 25 Sep 2021 | 9297 |  |
| Pre-Wave 4 | 26 Sep 2021 - 27 Nov 2021 | 771 | 573 [@ 215-217] |
| Wave-4 peak | 28 Nov 2021 - 22 Jan 2022 | 2000 | 3530 [@ 215-217] |
| Pre-Wave 5 | 23 Jan 2022 - 16 Apr 2022 | 2543 | 6 [@ 214-215], 148 [@ 215-217], 5 [@ 248-250] |
| Wave-5 peak | 17 Apr 2022 - 18 Jun 2022 | 3974 | 6 [@ 215-217], 2 [@ 248-250] |
