# Supplementary material for: Population-level toggling of T cell immune escape at human leukocyte antigen anchor residues in SARS-CoV-2 Spike proteins, in an ethnically diverse population region
Source: PLoS Comput Biol. 2025 Jul 21;21(7):e1013261. doi: 10.1371/journal.pcbi.1013261 (PMC12303384; doi:10.1371/journal.pcbi.1013261)
Supplement: S2 Table — Data were taken from an online source found at: Visualizing selection analysis results for evolution of nCOV (Nov 2021 update)/ Sergei Pond/ Observable (observablehq.com) and produced by Lytras et al (2022) [27]. (DOCX) [file pcbi.1013261.s002.docx]

**S2 Table.** ***GenBank Accession numbers for sarbecovirses used as a proxy for SARS-CoV-2 ancestral strains***

| KY770859.1 | OK017825.1 | OK017802.1 | OK017846.1 |
| --- | --- | --- | --- |
| MK211375.1 | OK017826.1 | OK017838.1 | OK017834.1 |
| MK211378.1 | OK017828.1 | OK017844.1 | OK017835.1 |
| KY417142.1 | OK017822.1 | OK017845.1 | OK017860.1 |
| OK017847.1 | OK017824.1 | OK017840.1 | OK017832.1 |
| OK017849.1 | OK017827.1 | OK017839.1 | DQ648857.1 |
| KC881005.1 | DQ412043.1 | OK017843.1 | OK017833.1 |
| MK211376.1 | KP886808.1 | OK017841.1 | OK017859.1 |
| KY417143.1 | KP886809.1 | OK017812.1 | MK211374.1 |
| OK017851.1 | KY770860.1 | OK017816.1 | DQ071615.1 |
| OK017848.1 | DQ412042.1 | OK017821.1 | KF569996.1 |
| OK017850.1 | KJ473812.1 | OK017820.1 | KU973692.1 |
| KY417150.1 | KJ473813.1 | OK017815.1 | JX993988.1 |
| KT444582.1 | KY938558.1 | OK017818.1 | OK017792.1 |
| OK017858.1 | KU182964.1 | OK017814.1 | OK017855.1 |
| KY417148.1 | KJ473811.1 | OK017817.1 | OK017856.1 |
| KC881006.1 | OK017794.1 | OK017813.1 | OK017853.1 |
| KF367457.1 | OK017799.1 | OK017819.1 | OK017857.1 |
| OK017831.1 | OK017795.1 | GQ153542.1 | OK017852.1 |
| OK017830.1 | OK017796.1 | GQ153543.1 | OK017854.1 |
| NC_014470.1 | OK017798.1 | DQ084199.1 | OK017793.1 |
| MZ190137.1 | OK017800.1 | DQ022305.2 | AY394995.1 |
| KY352407.1 | OK017797.1 | DQ084200.1 | FJ588686.1 |
| MT726045.1 | KF294457.1 | GQ153541.1 | KY417147.1 |
| MT726043.1 | OK017808.1 | GQ153539.1 | KJ473816.1 |
| MT726044.1 | OK017809.1 | GQ153547.1 | KY770858.1 |
| MZ190138.1 | OK017810.1 | GQ153540.1 | OK017829.1 |
| MW719567.1 | OK017811.1 | GQ153546.1 | KY417145.1 |
| KJ473814.1 | OK017836.1 | GQ153545.1 | KY417152.1 |
| OK017823.1 | OK017807.1 | GQ153544.1 | KY417144.1 |
| OK017842.1 | OK017837.1 | GQ153548.1 | MK211377.1 |
| KJ473815.1 | OK017801.1 | JX993987.1 | KY417146.1 |
|  |  |  | KY417149.1 |
|  |  |  | KY417151.1 |
