# Supplementary material for: Population-level toggling of T cell immune escape at human leukocyte antigen anchor residues in SARS-CoV-2 Spike proteins, in an ethnically diverse population region
Source: PLoS Comput Biol. 2025 Jul 21;21(7):e1013261. doi: 10.1371/journal.pcbi.1013261 (PMC12303384; doi:10.1371/journal.pcbi.1013261)
Supplement: S3 Table — HLA-I and corresponding 9-mer anchor residue motifs used (N = 137 unique HLA-motif pairs). The data was downloaded on 25 March 2022 from https://www.hiv.lanl.gov/content/immunology/motif_scan/motif_help.html#Motif_Scan_Help Four-digit HLA class I alleles with 9-mer anchor motifs containing at least two defined anchor positions were shortlisted (N = 137) and used to search for potential HLA binding peptides on the SARS-CoV-2 protein sequences. (DOCX) [file pcbi.1013261.s003.docx]

**S3 Table.** ***HLA-I and corresponding 9-mer anchor residue motifs used (N=137 unique HLA-motif pairs)***

| **HLA-I allele** | **9-mer Anchor residue motif** | **HLA-I allele** | **9-mer Anchor residue motif** | **HLA-I allele** | **9-mer Anchor residue motif** |
| --- | --- | --- | --- | --- | --- |
| A*02:01 | x[LM]xxxxxx[VL] | B*07:02 | x[P(V)]xxxxxx[L(F)] | C*01:02 | xx[P]xxxxx[L] |
| A*02:02 | x[L(A)]xxxxxx[LV] | B*07:03 | x[P(ND)]xxxxxx[L] | C*01:02 | x[AL]xxxxxx[L] |
| A*02:04 | x[L]xxxxxx[L] | B*07:05 | x[P]xxxxxx[L(F)] | C*01:03 | x[AL]xxxxxx[L] |
| A*02:05 | x[V(QL)]xxxxxx[L] | B*08:01 | xx[K(R)]x[K(RH)]xxxx | C*02:02 | x[A]xxxxxx[L] |
| A*02:06 | x[V(Q)]xxxxxx[V(L)] | B*08:02 | xx[K(RY)]x[K(H)]xxxx | C*02:03 | x[A]xxxxxx[L] |
| A*02:07 | x[L][D]xxxxx[L] | B*14:02 | x[R(K)]xx[R(H)]xxx[L] | C*03:02 | x[A]xxxxxx[FWY] |
| A*02:14 | x[QV]xxx[K]xx[VL] | B*15:02 | x[QLVP]xxxxxx[FYM] | C*03:03 | x[A]xxxxxx[LM] |
| A*02:14 | x[VQL(A)]xxxxxx[L(VM)] | B*15:03 | x[QK]xxxxxx[YF] | C*03:04 | x[A]xxxxxx[LM] |
| A*02:17 | x[L]xxxxxx[L] | B*15:08 | x[PA]xxxxxx[YF] | C*03:05 | x[A]xxxxxx[LM] |
| A*03:01 | x[LVM(IAST)]xxxxxx[KY(FR)] | B*15:09 | x[H]xxxxxx[LFM] | C*03:06 | x[A]xxxxxx[LM] |
| A*24:02 | x[YF]xxxxxx[FWIL] | B*15:10 | x[H]xxxxxx[L(F)] | C*03:07 | x[A]xxxxxx[LF] |
| A*26:01 | x[VTILF]xxxxxx[YF] | B*15:12 | x[Q(LM)]xxxxxx[YF] | C*03:08 | x[A]xxxxxx[LM] |
| A*26:02 | x[VTILF]xxxxxx[YFML] | B*15:13 | x[LIQVPM]xxxxxx[W] | C*03:09 | x[A]xxxxxx[LM] |
| A*26:03 | x[VFILT]xxxxxx[YFML] | B*15:16 | x[ST(F)]xxxxxx[YIVYF] | C*04:01 | x[YPF]xxxxxx[LFM] |
| A*29:02 | x[E(M)]xxxxxx[Y(L)] | B*15:17 | x[TS]xxxx[L]x[Y(F)] | C*04:02 | x[YP]xxxxxx[LF] |
| A*30:01 | x[YF(VLMIT)]xxxxxx[L(YFM)] | B*15:17 | x[TS]xxxxxx[YFLI] | C*04:03 | x[P]xxxxxx[LF] |
| A*30:02 | x[YFLV]xxxxxx[Y] | B*15:18 | x[H]xxxxxx[Y(F)] | C*04:04 | x[YP]xxxxxx[LF] |
| A*30:03 | x[FYIVL]xxxxxx[Y] | B*27:01 | x[RQ]xxxxxx[Y] | C*04:05 | x[YP]xxxxxx[LF] |
| A*32:01 | x[I]xxxxxxx[W] | B*27:02 | x[R]xxxxxx[FYILW] | C*04:06 | x[P]xxxxxx[LF] |
| A*66:01 | x[TV(APLIC)]xxxxxx[RK] | B*27:03 | x[R]xxxxxx[YF(RMWL)] | C*05:01 | x[A]xxxxxx[LF] |
| A*68:01 | x[VT]xxxxxx[RK] | B*27:04 | x[R]xxxxxx[YLF] | C*05:02 | x[A]xxxxxx[LF] |
| A*68:02 | x[TV]xxxxxx[VL] | B*27:05 | x[R]xxxxxx[LFYRHK(MI)] | C*06:03 | x[ALP]xxxxxx[L] |
| A*69:01 | x[VTA]xxxxxx[VL(MQ)] | B*27:06 | x[R]xxxxxx[L] | C*06:04 | x[RQ]xxxxxx[L] |
|  |  | B*27:07 | x[R]xxxxxx[LF] | C*07:01 | x[RHK]xxxxxx[Y] |
|  |  | B*27:09 | x[R]xxxxxx[LVFIM] | C*07:03 | x[YP]xxxxxx[YL] |
|  |  | B*27:10 | x[R]xxxxxx[YF] | C*07:04 | x[RQ]xxxxxx[LM] |
|  |  | B*35:01 | x[P(AVYRD)]xxxxxx[YFMLI] | C*07:05 | x[RQ]xxxxxx[Y] |
|  |  | B*35:03 | x[P(MILFVA)]xxxxxx[ML(F)] | C*07:06 | x[RHK]xxxxxx[Y] |
|  |  | B*35:05 | x[P]xxxxxx[F] | C*07:07 | x[RHK]xxxxxx[YL] |
|  |  | B*37:01 | x[DE(HPGSL)]xxxxx[FML(QKYL)][IL(TENDQGH)] | C*07:08 | x[RQ]xxxxxx[YL] |
|  |  | B*39:01 | x[RH]xxxxxx[L(VIM)] | C*07:09 | x[RHK]xxxxxx[YL] |
|  |  | B*39:02 | x[KQ]xxxxxx[L(FM)] | C*07:10 | x[YP]xxxxxx[FWY] |
|  |  | B*39:09 | x[RH(P)]xxxxxx[LF] | C*07:11 | x[R]xxxxxx[LM] |
|  |  | B*40:01 | x[E]xxxxxx[L] | C*07:12 | x[R]xxxxxx[LM] |
|  |  | B*40:02 | x[E]xxxxxx[IAVL] | C*08:01 | x[A]xxxxxx[LM] |
|  |  | B*40:06 | x[E(P)]xxxxxx[V(AP)] | C*08:02 | x[A]xxxxxx[LM] |
|  |  | B*42:01 | x[P]xxxxxx[L] | C*08:03 | x[A]xxxxxx[LM] |
|  |  | B*44:02 | x[E(MILD)]xxxxxx[FY] | C*08:04 | x[A]xxxxxx[LM] |
|  |  | B*44:03 | x[E(MILVD)]xxxxxx[YF] | C*08:05 | x[A]xxxxxx[LM] |
|  |  | B*46:01 | x[M(I)]xxxxxx[YF] | C*08:06 | x[A]xxxxxx[LM] |
|  |  | B*48:01 | x[QK(M)]xxxxxx[L] | C*12:02 | x[A]xxxxxx[FWY] |
|  |  | B*51:01 | x[APG(WF)]xxxxxx[VI(WMVL)] | C*12:03 | x[A]xxxxxx[FWY] |
|  |  | B*51:02 | x[APG]xxxxxx[IV] | C*12:04 | x[A]xxxxxx[L] |
|  |  | B*51:03 | x[APG(FW)]xxxxxx[VIF] | C*12:05 | x[A]xxxxxx[L] |
|  |  | B*53:01 | x[P]xxxxxx[WFL] | C*12:06 | x[A]xxxxxx[FWY] |
|  |  | B*56:01 | x[P]xxxxxx[A(L)] | C*14:02 | x[YP]xxxxxx[FWY] |
|  |  | B*57:01 | x[ATS]xxxxxx[FWY] | C*14:03 | x[YP]xxxxxx[FWY] |
|  |  | B*57:02 | x[ATS]xxxxxx[FW] | C*14:04 | x[YP]xxxxxx[FWY] |
|  |  | B*58:01 | x[AST(G)]xxxxxx[FW(Y)] | C*15:02 | x[A]xxxxxx[LMYF] |
|  |  | B*58:02 | x[ST]xxx[R]xx[F] | C*15:03 | x[A]xxxxxx[LMYF] |
|  |  | B*73:01 | x[R]xxxxxx[P] | C*15:04 | x[A]xxxxxx[L] |
|  |  | B*78:01 | x[PAG]xxxxx[A(KS)]x | C*15:05 | x[A]xxxxxx[L] |
|  |  | B*81:01 | x[P]xxxxxx[L] | C*15:06 | x[A]xxxxxx[LM] |
|  |  |  |  | C*15:07 | x[A]xxxxxx[LMY] |
|  |  |  |  | C*16:01 | x[A]xxxxxx[FWY] |
|  |  |  |  | C*16:02 | x[A]xxxxxx[L] |
|  |  |  |  | C*16:04 | x[A]xxxxxx[L] |
|  |  |  |  | C*17:01 | x[A]xxxxxx[L] |
|  |  |  |  | C*17:02 | x[A]xxxxxx[L] |
|  |  |  |  | C*18:01 | x[RQ]xxxxxx[LY] |
|  |  |  |  | C*1:802 | x[RQ]xxxxxx[LY] |
