# Supplementary material for: Population-level toggling of T cell immune escape at human leukocyte antigen anchor residues in SARS-CoV-2 Spike proteins, in an ethnically diverse population region
Source: PLoS Comput Biol. 2025 Jul 21;21(7):e1013261. doi: 10.1371/journal.pcbi.1013261 (PMC12303384; doi:10.1371/journal.pcbi.1013261)
Supplement: S4 Table — HLA-II and corresponding anchor residue motifs used (N = 45 unique HLA-motif pairs). The data were downloaded on 25 March 2022 from https://www.hiv.lanl.gov/content/immunology/motif_scan/motif_help.html#Motif_Scan_Help. HLA class II alleles with anchor motifs containing at least two defined anchor positions were shortlisted (N = 45) and used to search for potential HLA binding peptides on the SARS-CoV-2 protein sequences. (DOCX) [file pcbi.1013261.s004.docx]

**S4 Table.** ***HLA-II and corresponding anchor residue motifs used (N=45 unique HLA-motif pairs)***

| **HLA-II allele** | **Anchor residue motif (octamer or nonamer or decamer)** |
| --- | --- |
| DPA1*01:02/DPB1*02:01 | [FLMVWY]xxx[FLMY]xx[IAMV] |
| DPA1*01:03/DPB1*02:01 | [YLVFK]xx[DSQT]x[YFWV]xx[LVI] |
| DPA1*01:03/DPB1*02:01 | [FLM]xxx[FL]xx[IA] |
| DPA1*02:01/DPB1*04:01 | [FLYMIVA]xxxxx[FLYMVIA]xx[VYIAL] |
| DPA1*02:01/DPB1*09:01 | [RK]xxxx[AGL]xx[LV] |
| DQA1*01:02/DQB1*06:02 | xxxxx[LIV(APST)]xx[AGST(LIVP)] |
| DQA1*03:01/DQB1*03:02 | [RK]xxxx[AG]xx[NED] |
| DQA1*03:01/DQB1*03:02 | [TSW]xxxxxxx[RE] |
| DQA1*05:01/DQB1*02:01 | [FWYILV]xx[DELVIH]x[PDEHPA][DE]x[FWYILVM] |
| DQA1*05:01/DQB1*03:01 | [WYAVM]xx[A]x[AIVTS]xxx[QN] |
| DQB1*06:02 | [AFCILMNQSTVWYDE]x[AFGILMNQSTVWYCDE][AFGILMNQSTVWY]x[LIVAPST]xx[ASTGLIVP] |
| DRB1*01:01 | [YFWLIMVA]xx[LMAIVNQ]x[AGSTCP]xx[LAIVNFYMW] |
| DRB1*01:02 | [ILVM]xx[ALM]x[AGSTCP]xx[ILAMYW] |
| DRB1*03:01 | [LIFMV]xx[D]x[KR(EQN)]x[L][YLF] |
| DRB1*03:01 | [LIFMV]xx[D]x[KREQN]xx[YLF] |
| DRB1*04:01 | [FLV]xxxxxxx[NQST] |
| DRB1*04:01 | [FYWILVM]xx[FWILVADE]x[NSTQHR]xx[K] |
| DRB1*04:01 | [FYW]xxxxxxx[ST] |
| DRB4*01:01 | [FYW]xxxxxxx[ST] |
| DRB1*04:01 | [FYWILVM]xx[PWILVADE]x[NSTQHR][DEHKNQRSTYACILMV]x[DEHKNQRSTYACILMV] |
| DRB4*01:01 | [FYWILVM]xx[PWILVADE]x[NSTQHR][DEHKNQRSTYACILMV]x[DEHKNQRSTYACILMV] |
| DRB1*04:02 | [VILM]xx[YFWILMRNH]x[NSTQHK]x[RKHNQP]x[H] |
| DRB1*04:02 | [VILM]xx[YFWILMRN]x[NQSTK][RKHNQP]x[DEHLNQRSTYCILMVHA] |
| DRB1*04:04 | [VILM]xx[FYWILVMADE]x[NTSQR]xx[K] |
| DRB1*04:05 | [FYWVILM]xx[VILMDE]x[NSTQKD]xxx[DEQ] |
| DRB1*04:05 | [Y]xxxx[VT]xxx[D] |
| DRB4*01:01 | [Y]xxxx[VT]xxx[D] |
| DRB1*04:07 | [FYW]xx[AVTK]x[NTDS]xxx[QN] |
| DRB1*07:01 | [FYWILV]xx[DEHKNQRSTY]x[NST]xx[VILYF] |
| DRB1*11:01 | [WYF]xx[LVMAFY]x[RKH]xx[AGSP] |
| DRB1*11:01 | [YF]xxxx[RK]x[RK] |
| DRB3*02:02 | [YF]xxxx[RK]x[RK] |
| DRB1*11:04 | [ILV]xx[LVMAFY]x[RKH]xx[AGSP] |
| DRB1*12:01 | [ILFYV]x[LMNVA]xx[VYFINA]xx[YFMIV] |
| DRB1*13:01 | [ILVF]xx[YWLVAM]x[RK]xx[YFAST] |
| DRB1*13:01 | [ILV]xxxx[RK]xx[Y] |
| DRB3*01:01 | [ILV]xxxx[RK]xx[Y] |
| DRB1*13:02 | [YFVAI]xx[YWLVAM]x[RK]xx[YFAST] |
| DRB1*13:02 | [ILFY]xxxx[RK]xx[Y] |
| DRB3*13:01 | [ILFY]xxxx[RK]xx[Y] |
| DRB1*15:01 | [ILV]xxxxxxxx[HKR] |
| DRB5*01:01 | [ILV]xxxxxxxx[HKR] |
| DRB3*02:02 | [YFIL]xx[N]x[ASPDE]xx[LVISG] |
| DRB3*03:01 | [ILV]xx[N]x[ASPDE]xx[ILV] |
| DRB5*01:01 | [FYLM]xx[QVIM]xxxx[RK] |
