# Supplementary material for: Population-level toggling of T cell immune escape at human leukocyte antigen anchor residues in SARS-CoV-2 Spike proteins, in an ethnically diverse population region
Source: PLoS Comput Biol. 2025 Jul 21;21(7):e1013261. doi: 10.1371/journal.pcbi.1013261 (PMC12303384; doi:10.1371/journal.pcbi.1013261)
Supplement: S1 Text — (DOCX) [file pcbi.1013261.s008.docx]

## **S1 Text. Detailed methods for Phylogenetic inference of directionally evolving sites**

The direction of evolution on the phylogeny of protein sequences was determined to confirm whether the predicted immune escaping sites are under significant selective pressure and whether the predicted escape mutations are significant evolutionary targets. A Bayesian framework model FADE (FUBAR Approach to Directional Evolution), run within the HyPhy package v2.5.49 (Hypothesis testing using Phylogenies), was used to determine amino acid sites undergoing directional evolution(1-3). Directional evolution was determined within SARS-CoV-2 phylogeny and separately for the combined SARS-CoV-2/sarbecovirus phylogeny to understand evolutionary targets during infection spread in South Africa and related to zoonotic adaptation, respectively.

A single alignment for all SARS-CoV-2 sequences was produced by combining alignments across infection waves after removing 100% identical sequences resulting in N=10822 sequences without insertions. This SARS-CoV-2 alignment was combined with the 130 sarbecoviruses for a separate combined phylogeny.

**Phylogenetic inference**: A phylogenetic tree for the combined South Africa SARS-CoV-2 sequences was constructed using the neighbor joining method in HyPHy(3, 4). Predefined distance formulae based on the data, inference of phylogenetic distances using the number of substitutions observed per site in the data, and allowing for negative branch lengths were used. The tree was rooted using the ‘Ape’ and ‘Phylotools’ packages in R (v4.1.1)(5). To determine directional evolution during infection spread in South Africa, the phylogeny was rooted on WIV04. A second phylogenetic tree was constructed to estimate directional evolution related to zoonotic adaptation from past hosts into humans. For this, the phylogenetic tree was constructed from a combined alignment of South Africa SARS-CoV-2 sequences and the 130 sarbecoviruses proxy ancestral sequences. The combined SARS-CoV-2/sarbecovirus phylogeny was rooted on an estimated sarbecovirus ancestral sequence reconstructed from the 130 sarbecovirus nucleotide sequences using the ‘Ancestral sequence inference’ method (default settings) in MEGA with Wuhan-1 used as an outgroup(6-8).

**Directional evolution analysis**: The FADE model was run on each alignment and the corresponding rooted phylogenetic tree. The best fit amino acid substitution model (with the lowest AIC and highest likelihood) was first determined on the unrooted phylogenetic tree using the ‘AAModelComparison.bf’ within the HyPhy package(3). The parameters for model selection included fitting reversible models and assuming fixed substitution rates. In the final FADE run, the JTT and the default GTR models were used to estimate substitution rate matrices and branch lengths for the South Africa SARS-CoV-2 and SARS-CoV-2/sarbecovirus data, respectively(7). Default settings were used for the rest of the FADE parameters. Directional evolution at sites was determined across all branches in the South Africa SARS-CoV-2 data against the single WIV04 root. In the SARS-CoV-2/sarbecovirus data for evaluating directional evolution related to zoonosis, directional evolution was also modelled on the SARS-CoV-2 branches but using all sarbecovirus branches as evolutionary background against which the direction of evolution is determined. A Bayes Factor (BF) score of ≥100 in the FADE output was indicative of significant directional evolution towards the identified amino acid residue(s).

**REFERENCES**

1. Murrell B, Moola S, Mabona A, Weighill T, Sheward D, Kosakovsky Pond SL, et al. FUBAR: a fast, unconstrained bayesian approximation for inferring selection. Mol Biol Evol. 2013;30(5):1196-205.

2. Kosakovsky Pond SL, Poon AF, Leigh Brown AJ, Frost SD. A maximum likelihood method for detecting directional evolution in protein sequences and its application to influenza A virus. Mol Biol Evol. 2008;25(9):1809-24.

3. Pond SL, Frost SD, Muse SV. HyPhy: hypothesis testing using phylogenies. Bioinformatics. 2005;21(5):676-9.

4. Saitou N, Nei M. The neighbor-joining method: a new method for reconstructing phylogenetic trees. Mol Biol Evol. 1987;4(4):406-25.

5. Paradis E, Schliep K. ape 5.0: an environment for modern phylogenetics and evolutionary analyses in R. Bioinformatics. 2019;35(3):526-8.

6. Tamura K, Stecher G, Kumar S. MEGA11: Molecular Evolutionary Genetics Analysis Version 11. Mol Biol Evol. 2021;38(7):3022-7.

7. Jones DT, Taylor WR, Thornton JM. The rapid generation of mutation data matrices from protein sequences. Comput Appl Biosci. 1992;8(3):275-82.

8. Nei M, Kumar S. Molecular Evolution and Phylogenetics. Oxford University Press, New York.; 2000.
